# Supplementary material for: MetaQTL: a package of new computational methods for the meta-analysis of QTL mapping experiments
Source: BMC Bioinformatics. 2007 Feb 8;8:49. doi: 10.1186/1471-2105-8-49 (PMC1808479; doi:10.1186/1471-2105-8-49)
Supplement: Additional File 7 — MetaQTL Package : jar file and tutorial. This Zip archive contains both the MetaQTL JAR file and the files of the tutorial. [file 1471-2105-8-49-S7.zip › org.inra.metaqtl/doc/org/metaqtl/package-tree.html]

org.metaqtl Class Hierarchy


|  |  |  |
| --- | --- | --- |
| **Overview** \| **Package** \| Class \| Use \| **Tree** \| **Deprecated** \| **Index** \| **Help** | |  |
| PREV   **NEXT** | **FRAMES**    **NO FRAMES**     **All Classes** |  |


---


## Hierarchy For Package org.metaqtl

**Package Hierarchies:**: All Packages

---

## Class Hierarchy

- java.lang.Object
  - org.metaqtl.**ChromCluster**- org.metaqtl.**ChromCluster.ClusterEdge**- org.metaqtl.**ChromCluster.ClusterGraph**- org.metaqtl.**Chromosome**- org.metaqtl.**CMarkerSequence**- org.metaqtl.**EMCriteria** (implements java.io.Serializable)- org.metaqtl.**EMResult**- org.metaqtl.**MapCluster**- org.metaqtl.**MetaChrom**- org.metaqtl.**MetaDico**- org.metaqtl.**MetaMap**- org.metaqtl.**MetaQtlAnalysis**- org.metaqtl.**MetaQtlData**- org.metaqtl.**MetaQtlModel**- org.metaqtl.**MetaQtlResult**- org.metaqtl.**MultiFitResult**- org.metaqtl.**Qtl**- org.metaqtl.**Qtl.QTLCross** (implements java.io.Serializable)- org.metaqtl.**Qtl.QTLProj** (implements java.io.Serializable)- org.metaqtl.**Qtl.QTLStats** (implements java.io.Serializable)- org.metaqtl.**Qtl.QTLSupport** (implements java.io.Serializable)- org.metaqtl.**Qtl.QTLTrait** (implements java.io.Serializable)- org.metaqtl.**QtlPartition**- org.metaqtl.**Tree**- org.metaqtl.**TreeNode** (implements java.io.Serializable)

## Interface Hierarchy

- org.metaqtl.**IBioGenomeProperties**- org.metaqtl.**IBioLGroupProperties**- org.metaqtl.**IBioLocusProperties**- org.metaqtl.**IMetaAlgorithm**- org.metaqtl.**IMetaQtlConstants**

---


|  |  |  |
| --- | --- | --- |
| **Overview** \| **Package** \| Class \| Use \| **Tree** \| **Deprecated** \| **Index** \| **Help** | |  |
| PREV   **NEXT** | **FRAMES**    **NO FRAMES**     **All Classes** |  |


---
